# Lifes essential 8 score and 10-year cardiovascular outcomes in atrial fibrillation: A UK biobank analysis with simulated lifestyle improvement

**DOI:** 10.1016/j.ajpc.2025.101399

**Published:** 2025-12-29

**Authors:** Charlotte J. Fitzhugh, Helen Jones, Lawrence Foweather, Benjamin J.R. Buckley

**Affiliations:** a Research Institute of Sport and Exercise Sciences, Liverpool John Moores University, Liverpool, United Kingdom; b Liverpool Centre for Cardiovascular Science at University of Liverpool, Liverpool John Moores University, and Liverpool Heart & Chest Hospital, Liverpool, United Kingdom

**Keywords:** Atrial fibrillation, Cardiovascular health, Life's Essential 8, Lifestyle modification, Mortality, Major adverse cardiovascular events

## Abstract

**Background:**

Atrial fibrillation (AF) is associated with high risks of mortality and cardiovascular events, yet the prognostic value of comprehensive lifestyle and clinical health metrics remains uncertain.

**Objective:**

To investigate whether cardiovascular health (CVH), as measured by the American Heart Association's Life’s Essential 8 (LE8) score, is associated with clinical outcomes in people with AF, and to estimate the impact of simulated improvement in CVH components.

**Methods:**

Data were drawn from the UK Biobank, a prospective population-based cohort. Participants with AF were identified using ICD codes. CVH was assessed using a modified LE8 score (range 0–100), derived from smoking status, cholesterol, blood pressure, BMI, HbA1c, physical activity, diet, and sleep. Primary outcomes were all-cause mortality and major adverse cardiovascular events (MACE: ischaemic heart disease, myocardial infarction, stroke, and heart failure). Associations were analysed using Cox models with penalised splines, presented in Kaplan-Meier curves. Population attributable and potential impact fractions were estimated.

**Results:**

Among 23,758 individuals with AF and 10-year follow-up, higher CVH scores associated with lower risk of all-cause mortality and MACE in a non-linear, graded pattern. Compared with the lowest quartile, the highest CVH quartile had 39% lower risk of all-cause mortality (HR: 0.61, 95% CI: 0.56–0.67) and 38% lower risk of MACE (HR: 0.62, 95% CI: 0.58–0.67; both *p* < 0.001). Associations were modified by age and multimorbidity. Simulated improvements in CVH could reduce all-cause mortality by 10% and MACE by 7%, with diet, smoking, blood pressure, and BMI contributing most.

**Conclusion:**

Higher LE8 scores were independently associated with lower all-cause mortality and MACE risk in people with AF, supporting the role of lifestyle-based secondary prevention in AF care.

## 1 Background

Atrial Fibrillation (AF) is the most common cardiac arrhythmia, affecting 59 million individuals worldwide, with prevalence expected to rise to 6—12 million people in the United States and 18 million in Europe within the next 25—35 years [1,2]. AF-related symptoms can be disabling and highly variable, including palpitations, breathlessness, fatigue, dizziness, and reduced exercise tolerance [3]. The condition typically progresses from paroxysmal to more persistent forms if left untreated, particularly in the presence of cardiovascular disease risk factors and comorbidities [4]. AF is strongly associated with hypertension, obesity and impaired glucose control, as well as poor mental health, reduced quality of life, and increased mortality rates [5].

Lifestyle behaviours and cardiometabolic risk factors, such as poor diet, physical inactivity, obesity, smoking, and elevated blood pressure are key contributors to cardiovascular diseases (CVD) and adverse outcomes in individuals with AF. Addressing these modifiable risk factors is central to both primary and secondary prevention efforts. In response, the American Heart Association (AHA) developed ‘Life’s Simple 7′ (LS7), a cardiovascular health metric, combining four behavioural (physical activity, diet, body mass index [BMI], and smoking status) and three biological measures (blood pressure, total cholesterol, and fasting blood glucose) (7). Recognising the importance of sleep for cardiovascular outcomes, this framework was updated in 2022 to form the ‘Life’s Essential 8’ (LE8) score (8). LE8 is a composite score ranging from 0 to 100, with higher values reflecting better cardiovascular health across modifiable domains. Unlike traditional risk calculators that estimate future disease risk based on the presence of clinical risk factors, the LE8 captures upstream lifestyle and metabolic factors (8). Epidemiological studies have observed an elevated risk of a range of long-term health conditions, including chronic kidney disease, stroke, dementia, hypertension, and atrial fibrillation, with lower LE8 scores [[6], [7], [8], [9]]. In a UK Biobank study of 250,824 participants with a median follow-up of 10.4 years, those in the lowest quartile of cardiovascular health scores had a two-fold higher risk of major adverse cardiovascular events (MACE: including ischaemic heart disease. Myocardial infarction, stroke and heart failure) than those in the highest quartile [10]. However, no study to date has evaluated the prognostic utility of the LE8 score in individuals with established AF, representing a critical gap in our understanding of lifestyle factors in secondary prevention.

Observational studies have shown that better LS7 scores are associated with a lower incidence and burden of AF. For example, in the ARIC cohort (*n* = 2363), each 1-point increase in LS7 was associated with a 13 % reduction in the odds of continuous AF, while low physical activity, higher BMI, and elevated fasting glucose were independently associated with greater AF burden [11]. Another large cohort study (*n* = 9576) found that participants in the optimal LS7 category (defined as a score between 10—14) had 32 % lower odds of developing AF compared to those in the lowest category, with each additional point associated with a 5 % reduction in risk [12].

This gap is particularly timely to address, given that a 2024 Cochrane review demonstrated significant reductions in AF severity, burden, and recurrence with comprehensive exercise-based cardiac rehabilitation for people with AF [13,14]. However, meta-analyses of 20 randomised clinical trials were underpowered to investigate the impact on clinical events [13,14]. Given the strong associations between AF, comorbidities, and modifiable lifestyle factors, there is a clear need to evaluate whether comprehensive health metrics such as LE8 can predict long-term outcomes. If shown to be predictive, these metrics could help guide lifestyle-based interventions within integrated care pathways for AF (16). Therefore, this study aimed to evaluate whether LE8 scores are associated with long-term risks of all-cause mortality and MACE in individuals with AF, and to estimate the potential impact of simulated improvements in LE8 scores—overall and for individual components—on these outcomes.

## 2 Methods

The UK Biobank is a population-based prospective cohort study that recruited 502,655 UK residents aged 40–69 years between 2006 and 2010, with ongoing follow-up [15]. Participants provided written informed consent for the use of their biological samples, data linkage to electronic health records, and future re-contact. Ethical approval was granted by the Northwest Multicentre Research Ethics Committee. Because data were de-identified, this study did not require additional approval. The UK Biobank provides comprehensive health information, including detailed medical diagnoses and lifestyle habits. At baseline, participants completed questionnaires on demographics, lifestyle, and medical history, and underwent physical measurements. For ongoing follow-up, the UK Biobank has been collecting incident disease diagnoses through linkage of multiple national datasets, including primary care records, hospital inpatient and outpatient data, and death registrations [15]. Further information about the UK Biobank is available on the official website: https://ukbiobank.ac.uk/.

### 2.1 Cardiovascular health score

***Life’s Essential 8 Score.*** The adapted LE8 score was defined according to AHA criteria [16], consisting of four behavioural (physical activity, diet quality, exposure to cigarette smoking, and sleep) and four biological measures (BMI, blood lipids, blood glucose, and blood pressure) (Supplementary Table 1). Due to differences in dietary data collected by UK Biobank, we derived an adapted diet score based on nine available food items (processed meat, red meat, total fish, alcohol, spread type, cereal intake, salt added to food, water, fruit and vegetables), rather than the DASH-style eating pattern originally proposed by the AHA [17]. (See Supplementary Table 2 for full details). Each component was scored from 0 to 100, with the overall LE8 calculated as the mean of the eight components, ranging from 0 (poorest CVH) to 100 (optimal CVH). Full thresholds, UK Biobank field IDs, and coding rules are provided in Supplementary Table S1–S3 [16].

### 2.2 Measures

**Atrial Fibrillation.** AF was defined using hospital admissions and death registry data, identified by ICD-10 code I48. Diagnoses were ascertained via linkage to Hospital Episode Statistics (HES; England and Wales) and Scottish Morbidity Records (SMR01; Scotland). Only participants with confirmed AF before or at baseline were included. Self-report, Read codes, and ICD-9 data were not used; cases were restricted to ICD-10 coding to ensure consistency and reproducibility.

***Outcomes.*** The primary outcomes were all-cause mortality and a composite of major adverse cardiovascular events (MACE), defined as first occurrence of ischaemic heart disease (IHD: I20–I25), myocardial infarction (I21–I23), stroke (I60, I61, I63, I64), or heart failure (I50.0, I50.1, I50.9). The date of death was obtained from death certificates (NHS Information Centre, England/Wales; NHS Central Register, Scotland). Hospital admissions were captured through linkage with HES and SMR01. Details of data linkage are available at http://content.digital.nhs.uk/services

***LE8 Variables and Covariates.*** Smoking status was self-reported (current, former, never). BMI was calculated from measured height and weight. Physical activity was assessed via adapted short-form IPAQ [18]. The adapted diet score (above) was calculated and then converted into LE8 quartiles (Supplementary Table S1). Sleep was self-reported as average hours per 24 h (including naps). Non-HDL cholesterol and HbA1c were measured from baseline blood samples; blood pressure was measured using an automated device. Covariates included age, sex, and socioeconomic status (Townsend deprivation index[19]). All LE8 scores were derived at baseline and not updated during follow-up.

### 2.3 Statistical analyses

Descriptive baseline characteristics were summarised by LE8 quartiles as means (SD) for continuous variables and frequencies (percentages) for categorical variables. Complete case analysis was undertaken with participants with missing data for the LE8 components excluded prior to analysis. For multivariable analyses, a complete-case approach was used, whereby participants with missing data for covariates included in Models 2 and 3 were excluded. No imputation procedures were applied due to minimal missing covariate data (<1 %).

Associations between continuous LE8 scores and outcomes (all-cause mortality and MACE) were examined using Cox proportional hazard models with penalised splines. Penalised cubic splines were fitted using the pspline() function from the *survival* package, with spline complexity specified a priori using 4 degrees of freedom, consistent with our prior epidemiological work, allowing sufficient flexibility and limiting risk of overfitting. Sensitivity analyses were conducted to assess robustness to alternative spline specifications. We fitted three models: Model 1 unadjusted; Model 2 adjusted for age, sex, and Townsend deprivation index; and Model 3 further adjusted for comorbidities (acute ischaemic stroke, acute MI, chronic coronary syndrome, heart failure, type 2 diabetes, and depression). Multimorbidity was defined as the presence of ≥1 of these pre-specified cardiometabolic comorbidities at baseline. To aid interpretability, LE8 scores were also divided into quartiles. Cox proportional hazard models investigating associations between LE8 quartile and all-cause mortality and MACE. Individuals in the lowest quartile (least healthy) were used as the reference group. The results are reported as hazard ratios (HR) with 95 % confidence intervals (95 % CI). Effect modification (moderation) analyses were performed to assess whether the association between LE8 score and outcomes varied across age, sex, and multimorbidity strata. Moderation was evaluated on the multiplicative scale by including an interaction term between LE8 (continuous, modelled using penalised splines) and each moderator within Cox proportional hazards models, and comparing models with and without interaction using likelihood ratio tests. Interaction terms were specified between the spline-based LE8 function and each moderator variable. Significant interactions were interpreted as evidence that the relative association (hazard ratio function) between LE8 and outcomes differed between subgroups (i.e., different slopes/shapes), rather than implying additive effects on an absolute risk scale. Significant global interactions were then explored and visualised using Likelihood ratio tests and stratified Kaplan-Meier curves were presented to display interaction effects. Proportional hazards assumptions were assessed using Schoenfeld residuals and visual inspection of log–log survival plots, and no substantial violations were observed.

The population attributable fraction (PAF) was estimated to calculate the proportion of mortality and MACE events that could be attributed to LE8 scores, assuming a causal relationship [20]. This PAF was estimated based on the adjusted HR derived from the nonlinear associations. The potential impact fractions (PIFs) of a hypothetical scenario were also calculated, under the assumption that the effect of improving the LE8 score was constant across the range of LE8 values [21]. We simulated a targeted intervention approach by modelling a 20-point increase in the LE8 score for all individuals with a score below 50 and estimated the potential impact on all-cause mortality and MACE. Additional PIF analyses were conducted to estimate the individual contribution of each LE8 component to these outcomes.

Both PAF and PIF estimates should be interpreted as hypothetical, scenario-based measures. These analyses rely on observational, baseline-only data and therefore assume no residual confounding, accurate exposure measurement, and stability of cardiovascular health behaviours over follow-up. In addition, PAFs derived from penalised spline-based hazard functions are inherently model-dependent and intended to summarise population-level patterns rather than provide precise causal effect estimates.

Two sensitivity analyses were performed: (1) a 2-year landmark analysis excluding participants with events within the first two years of follow-up [22], and (2)a competing risk analysis was performed for MACE accounting for all-cause mortality as a competing risk [23]. All analyses were conducted using RStudio (version 2024.12.1 + 563), with statistical significance set at *p* < 0.05.

## 3 Results

### 3.1 Baseline characteristics

After excluding participants with missing data, 23,758 participants with AF were included in the analyses (Supplementary Figure 1). Table 1 presents the baseline characteristics stratified by quartiles of the LE8 score, with the first quartile representing the lowest lifestyle score and the fourth quartile representing the highest. The mean age of participants was 61 years (SD = 6).

**Table 1:** General cohort characteristics at baseline of participants included by quartiles of the LE8 score.

	1st quartile (Least healthy)	2nd quartile	3rd quartile	4th quartile (Healthiest)	Total
n	5940	5940	5939	5939	23,758
Baseline age (years), mean (SD)	61.91 (5.9)	62.16 (5.9)	61.84 (6.2)	61.37 (6.4)	61.82 (6.1)
Female ( %)	1296 (22 %)	1662 (28 %)	2126 (36 %)	2729 (46 %)	7813 (33 %)
Deprivation index, mean (SD)	−0.7 (3.3)	−1.3 (3.1)	−1.6 (2.9)	−1.9 (2.8)	−1.4 (3.1)
BMI (SD)	32.72 (5.6)	29.65 (4.7)	27.64 (3.9)	25.22 (3.1)	28.81 (5.2)
SBP (SD)	150.81 (19.5)	147.53 (19.5)	144.47 (19.3)	135.37 (19.3)	144.55 (20.2)
DBP (SD)	86.23 (11.8)	84.47 (11.1)	82.76 (10.4)	78.23 (9.7)	82.92 (11.2)
Physical activity (MET-min/week), mean (SD)	1830.8 (2534.0)	2753.1 (2808.8)	3054.6 ± 2832.0	3108.0 (2626.3)	2686.6 (2751.1)
Smoking Current ( %)	1444 (24 %)	709 (12 %)	226 (4 %)	36 (1 %)	2415 (10 %)
Smoking Previous ( %)	3516 (59 %)	3427 (58 %)	2679 (45 %)	1061 (18 %)	10,683 (45 %)
Smoking Never ( %)	917 (15 %)	1791 (30 %)	3027 (51 %)	4842 (82 %)	10,577 (45 %)
HbA1c (mmol/mol) Cholesterol	40.6 ± 10.3	37.8 (7.4)	36.6 (6.8)	35.7 (5.4)	37.7 ± 7.9
LDL (mmol/L)	3.2 (0.9)	3.3 (0.9)	3.4 (0.9)	3.4 ± 0.8	3.3 (0.9)
HDL(mmol/L)	1.2 (0.3)	1.3 (0.4)	1.4 (0.4)	1.5 (0.4)	1.4 (0.4)
Antihypertensives ( %)	2664 (45 %)	1839 (31 %)	1166 (20 %)	629 (11 %)	6298 (27 %)
Cholesterol Medication	3060 (52 %)	2019 (34 %)	1385 (23 %)	633 (11 %)	7097 (30 %)
Insulin	221 (4 %)	80 (1 %)	55 (1 %)	18 (0 %)	374 (2 %)

N: number; SD: standard deviation, BMI: Body Mass Index; SBP: systolic blood pressure, DBP, diastolic blood pressure, HbA1c: glycated haemoglobin, LDL: Low-density lipoprotein, HDL: High-density lipoprotein.

Overall, individuals in the highest quartile were more likely to be female and had lower BMI and blood pressure, higher physical activity levels, and better glycaemic control. In contrast, those in the lowest quartile had higher rates of smoking, lower physical activity levels, elevated blood pressure, and poorer metabolic profiles, including higher HbA1c levels. Socioeconomic factors also showed a clear gradient, with individuals in quartile 4 having lower deprivation index scores compared to those in quartile 1.

### 3.2 Nonlinear analysis of LE8 score and mortality

**Fig. 1:**
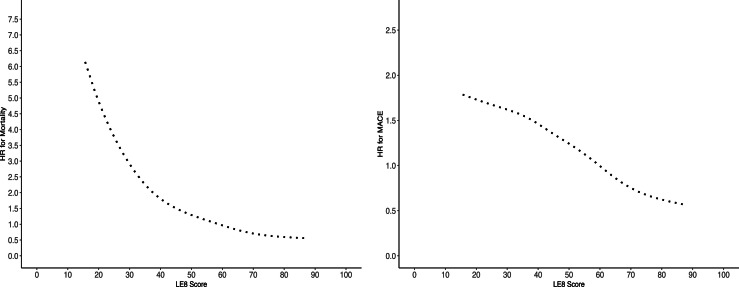
HRs for mortality (A) and MACE (B) across Life’s Essential 8 score using a non-linear cox hazards regression analysis with penalized splines.

Nonlinear analysis was conducted to examine the association between the LE8 score (as a continuous variable) and all-cause mortality using Cox proportional hazard models fitted with a penalized spline. In the unadjusted model, a higher LE8 score was associated with a significantly lower risk of all-cause mortality (β = −0.031, SE = 0.001, *p* < 0.001). To account for potential confounding, two additional models were fitted. Model 2 adjusted for age, sex, and the Townsend deprivation index. The association remained significant (β = −0.028, SE = 0.001, *p* < 0.001). Model 3 further adjusted for comorbidities, including acute ischaemic stroke, acute myocardial infarction, chronic coronary syndrome, heart failure, type 2 diabetes mellitus, and depression. The association with all-cause mortality remained significant (β = −0.025, SE = 0.001, *p* < 0.001). Associations between the continuous LE8 score and risk of all-cause mortality are shown in Fig. 1.

### 3.3 Nonlinear analysis of LE8 score and MACE

Nonlinear analysis was also conducted to examine the association between the continuous LE8 score and MACE using Cox proportional hazard models with penalized splines. In the unadjusted model, a significant nonlinear inverse association was observed between LE8 score and MACE risk (β = −0.022, SE = 0.001, *p* < 0.001), indicating that individuals with higher LE8 scores had a significantly lower risk of MACE. This association remained statistically significant in Model 2 (adjusted for age, sex, and Townsend deprivation index; β = −0.019, SE = 0.001, *p* < 0.001) and Model 3 (further adjusted for comorbidities; β = −0.018, SE = 0.001, *p* < 0.001). Associations between the continuous LE8 score and risk of MACE are shown in Fig. 1. Crude event rates for all-cause mortality and MACE are presented in Table 2, Table 3, respectively.

**Table 2:** Event Rates for Mortality Across LE8 Quartiles.

LE8 Quartile	Deaths (n)	Person-Years	Event Rate	Rate per 1000 Person-Years	95 % CI
1 (Lowest)	1577	34,142	0.04	46	44–49
2	1178	34,758	0.03	34	32–36
3	987	35,195	0.02	28	26–30
4 (Highest)	775	35,747	0.02	22	20–23

**Note.** Event rate represents the proportion of deaths per person-year. The rate per 1000 person-years is presented with 95 % confidence intervals (CI).

**Table 3:** Event Rates for Major Adverse Cardiovascular Events (MACE) Across LE8 Quartiles.

LE8 Quartile	MACE Events	Person-Years	Event Rate	Rate per 1000 Person-Years	95 % CI
1 (Lowest)	1812	28,565	0.06	63	61–66
2	1501	28,778	0.05	52	50–55
3	1310	29,285	0.04	45	42–47
4 (Highest)	1071	29,830	0.03	36	34–38

**Note.** Event rate represents the proportion of deaths per person-year. The rate per 1000 person-years is presented with 95 % confidence intervals (CI).

### 3.4 Population attributable fraction

A population attributable fraction (PAF) was calculated, incorporating all components contributing to the overall LE8 score. The estimated population attributable fraction suggested that 40 % (95 % CI: 38–42) of all-cause mortality and 32 % (95 % CI: 30–34) of MACE in this AF cohort were attributable to suboptimal LE8 levels under the modelling assumptions.

### 3.5 Potential impact fraction analysis

A potential impact fraction (PIF) was estimated to explore the potential clinical benefits of lifestyle improvement. By simulating a 20-point increase in the LE8 score—representing a meaningful improvement in one lifestyle behaviour—among individuals with an LE8 score below 50, we estimated a 10 % reduction in all-cause mortality (95 % CI: 0.09–0.10) and a 7 % reduction in MACE (95 % CI: 0.06–0.08), at the population level.

To explore the individual contributions of LE8 components and their relative impact on all-cause mortality and MACE, we conducted separate PIF analyses for each lifestyle and biological variable. This approach allowed estimation of the potential reduction in adverse events associated with improving a single component by 20 points among individuals with suboptimal baseline scores, defined as <50 for smoking, diet, and blood pressure; <60 for non-HDL cholesterol and HbA1c; and <70 for BMI, physical activity, and sleep (see supplementary data for threshold values).

For all-cause mortality, the largest estimated reductions were observed with dietary improvement, which was associated with a 6.6 % reduction (95 % CI: 0.05–0.09); smoking cessation, with a 6.4 % reduction (95 % CI: 0.06–0.07); and blood pressure control, with a 4.3 % reduction (95 % CI: 0.03–0.05). Smaller estimated reductions were seen for sleep improvement: 1.6 % reduction (95 % CI: 0.01–0.02); increased physical activity: 1.6 % reduction (95 % CI: 0.01–0.02); and BMI improvement: 1.4 % reduction (95 % CI: 0.01–0.02). Minimal reductions were associated with improved non-HDL cholesterol: 0.02 % reduction (95 % CI: 0.00–0.04), and no reduction was observed for HbA1c: 0 % reduction (95 % CI: 0.00–0.00). A full summary of all-cause mortality-related PIF estimates by LE8 component is presented in Table 4.

**Table 4:** Population Impact Fractions (PIFs) and 95 % Confidence Intervals for Mortality and MACE by LE8 component.

LE8 Component	Mortality PIF (95 % CI)	MACE PIF (95 % CI)
Diet	6.6 (5–9)	4 (3–5)
Smoking	6.4 (6–7)	4 (2–6)
Blood Pressure	4.3 (3–5)	3.7 (3–4)
Physical Activity	1.6 (1–2)	1.3 (1–2)
Sleep	1.6 (1–2)	1.5 (1–2)
BMI	1.4 (1–2)	1.1 (1–1)
Non-HDL Cholesterol	0.02 (0.0–0.04)	0.03 (0.0–0.05)
HbA1c	0.00 (0.0–0.0)	0.00 (0.0–0.0)

**Note.** PIF, Potential Impact Fraction. Values represent the estimated percentage reduction in mortality associated with a 20-point improvement in each LE8 component among individuals below threshold scores. 95 % confidence intervals (CI) are shown.

For MACE, the greatest estimated reductions were associated with dietary improvement: 4.0 % reduction (95 % CI: 0.02–0.06); blood pressure control: 4.0 % reduction (95 % CI: 0.03–0.05); and BMI improvement: 3.7 % reduction (95 % CI: 0.03–0.04). Smaller reductions were associated with smoking: 1.5 % reduction (95 % CI: 0.01–0.02); sleep: 1.3 % reduction (95 % CI: 0.01–0.02); and physical activity: 1.1 % reduction (95 % CI: 0.01–0.01). Improvements in non-HDL cholesterol led to a small reduction of 0.03 % (95 % CI: 0.00–0.05), while HbA1c showed no measurable effect: 0 % reduction (95 % CI: 0.00–0.00) (Table 4).

### 3.6 Association between LE8 quartiles and mortality

**Fig. 2:**
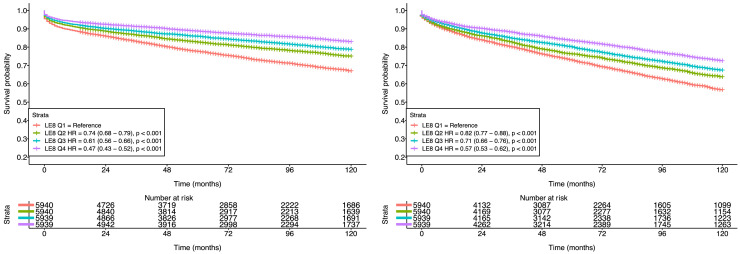
Kaplan-Meier curves presenting survival probability for all-cause mortality (A) and MACE (B) stratified by quartiles of Life’s Essential 8.

When stratifying LE8 scores into quartiles, Cox proportional hazard models demonstrated a strong inverse association between higher LE8 scores and all-cause mortality. In Model 1 (unadjusted), higher quartiles of LE8 were associated with significantly lower risk of all-cause mortality. Compared to the reference group (Q1), participants in Q2 had a 26 % lower risk of all-cause mortality (HR: 0.74, 95 % CI: 0.68–0.79, *p* < 0.001). The protective association was stronger in Q3 (HR = 0.61, 95 % CI: 0.56–0.66, *p* < 0.001) and Q4 (HR = 0.47, 95 % CI: 0.43–0.52, *p* < 0.001) (Fig. 2).

In Model 2, after adjusting for age, sex, and the Townsend deprivation index, the association remained significant, though slightly attenuated. Compared to Q1, participants in Q2 had a 25 % lower risk of mortality (HR: 0.75, 95 % CI: 0.70–0.81, *p* < 0.001), those in Q3 had a 36 % lower risk (HR: 0.66, 95 % CI: 0.60–0.71, *p* < 0.001), and those in Q4 had a 47 % lower risk (HR: 0.53, 95 % CI: 0.49–0.58, *p* < 0.001).

Model 3, which further adjusted for pre-existing comorbidities, continued to demonstrate a statistically significant inverse association. Compared to individuals in Q1, those in Q2 had a 19 % lower risk (HR: 0.81, 95 % CI: 0.75–0.87, *p* < 0.001), Q3 had a 27 % lower risk (HR: 0.73, 95 % CI: 0.67–0.79, *p* < 0.001), and Q4 had a 39 % lower risk of all-cause mortality (HR: 0.61, 95 % CI: 0.56–0.67, *p* < 0.001).

### 3.7 Association between LE8 quartiles and MACE

Cox proportional hazard models demonstrated a significant inverse association between higher LE8 quartiles and MACE. In Model 1 (unadjusted), compared to the reference category (Q1), participants in Q2 had an 18 % lower risk of MACE (HR = 0.82 (95 % CI: 0.77–0.88, *p* < 0.001), Q3 had a 29 % lower risk of MACE (HR = 0.71, 95 % CI: 0.66–0.76, *p* < 0.001), and Q4 had a 43 % lower risk of MACE (HR = 0.57, 95 % CI: 0.53–0.62, *p* < 0.001) (Fig. 2).

In Model 2, after adjusting for age, sex, and Townsend deprivation index, the associations remained statistically significant. Individuals in Q2 had a 17 % lower risk of MACE compared to those in Q1 (HR = 0.83, 95 % CI: 0.78–0.89, *p* < 0.001). A stronger reduction in risk was observed for Q3, with a 26 % lower risk (HR = 0.74, 95 % CI: 0.69–0.79, *p* < 0.001). The greatest risk reduction was seen in Q4, with individuals showing a 39 % lower risk compared to Q1 (HR = 0.61, 95 % CI: 0.57–0.66, *p* < 0.001).

Further adjustment for comorbidities in Model 3 yielded similar results. Individuals in Q2 had a 16 % lower risk of MACE (HR = 0.84, 95 % CI: 0.73–0.90, *p* < 0.001). Risk reductions were more pronounced in higher quartiles, with a 26 % lower risk in Q3 (HR = 0.74, 95 % CI: 0.69–0.81, *p* < 0.001), and a 38 % lower risk in Q4 (HR = 0.62, 95 % CI: 0.58–0.67, *p* < 0.001).

Significant moderation effects were observed in the relationship between LE8 and both all-cause mortality and MACE for age, sex, and multimorbidity. For all-cause mortality, age (χ² = 205.11, *p* < 0.001), sex (χ² = 22.53, *p* = 0.027 for females; χ² = 21.55, *p* = 0.036 for males), and multimorbidity (χ² = 151.02, *p* < 0.001 for those without morbidity; χ² = 648.42, *p* < 0.001 for those with morbidity) significantly moderated the association between LE8 and survival. Higher LE8 levels were associated with improved survival across all subgroups.

**Fig. 3:**
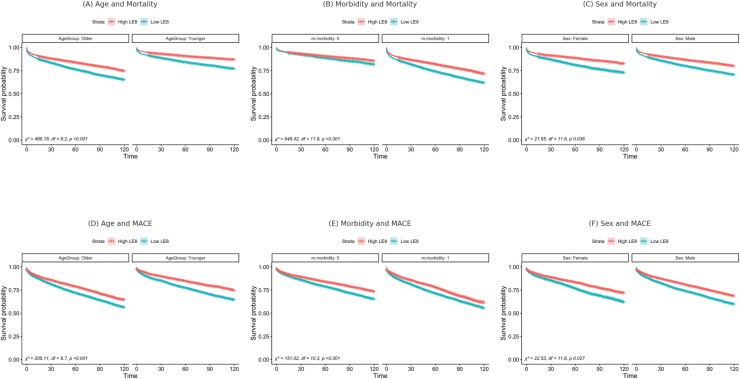
Kaplan-Meier curves presenting survival probability for Mortality stratified by Age (A), Morbidity (B), Sex (C), and MACE stratified by Age (D), Morbidity (E), Sex (F), for both high and low LE8 score (above and below median).Sensitivity Analyses.

Similarly, for MACE, age (χ² = 485.18, *p* < 0.001), sex (χ² = 22.53, *p* = 0.027 for females; χ² = 21.55, *p* = 0.036 for males), and multimorbidity (χ² = 151.02, *p* < 0.001 for individuals without morbidity; χ² = 648.42, *p* < 0.001 for individuals with morbidity) significantly moderated the relationship between LE8 and event-free survival. These interactions indicate differences in the strength of association between LE8 score and outcomes across subgroups, rather than large differences in absolute risk. Stratified Kaplan-Meier curves illustrating these moderation effects for mortality and MACE are presented in Fig. 3.

To assess the robustness of the findings, we performed a two-year landmark analysis, excluding participants who experienced events within the first two years of follow-up. The results indicated that the relationship between LE8 score, and all-cause mortality remained consistent. Secondly, a competing risk analysis was performed to evaluate the association between the LE8 quartiles and MACE, accounting for all-cause mortality as a competing risk. The results indicated that the relationship between LE8 score, and the risk of MACE was not impacted when factoring for mortality as a competing risk. Details of these analyses are available in the supplementary materials.

## 4 Discussion

In this large, population-based prospective cohort study, we observed for the first time that greater adherence to cardiovascular health behaviours - as captured by the LE8 score - was associated with significantly lower risks of all-cause mortality and MACE in people with AF. Specifically, those in the highest LE8 quartile had a 39 % lower risk of all-cause mortality and a 38 % lower risk of MACE compared to those in the lowest quartile. Higher LE8 scores were consistently linked to lower risk across all covariate and sensitivity models, with older age, sex, and multimorbidity modifying these associations (Fig. 3). Notably, the presence of multimorbidity exacerbated the risks associated with lower LE8 scores, suggesting that individuals with multiple health conditions may derive the greatest benefit from lifestyle improvement.

Our simulation analyses provide novel insights into how modifiable lifestyle factors influence prognosis in AF and may support the use of the LE8 score as a risk stratification tool to guide targeted lifestyle interventions in this high-risk population. The PAF analyses suggested that a substantial proportion of all-cause mortality and MACE events may be attributable to suboptimal cardiovascular health, as captured by the LE8 score, under the modelling assumptions. The estimated contributions of individual LE8 components differed, with the largest potential reductions in adverse outcomes observed in association with improvements in diet, blood pressure, BMI, and smoking cessation. These findings are consistent with evidence from the Atherosclerosis Risk in Communities Study, which, although focused on AF burden rather than clinical events, identified low physical activity level, high BMI, and high fasting blood glucose as being associated with the greatest AF burden [11].

Findings from interventional studies further support targeting behaviours such as diet, blood pressure, BMI, and smoking cessation. The PREDIMED-Plus Trial demonstrated that an intensive lifestyle program incorporating dietary changes reduced markers of systemic inflammation, and improved metabolic profiles—mechanisms relevant to AF development and progression [24]. The LEGACY study found that structured weight loss significantly reduced AF burden and symptom severity, with ≥10 % weight loss associated with a sixfold increase in arrhythmia-free survival [25]. Similarly, the ARREST-AF trial demonstrated that intensive risk factor modification—including weight loss, physical activity, blood pressure control, and diabetes management—significantly reduced AF recurrence rates following catheter ablation [26].

A Cochrane review of 20 trials demonstrated that exercise-based cardiac rehabilitation (ExCR) was associated with reduced AF recurrence, severity, and burden while improving quality of life and functional capacity compared with controls [27]. ExCR typically incorporates structured exercise sessions, cardiovascular risk factor management, lifestyle and dietary counselling, stress reduction strategies, and psychosocial support [28]. Collectively, these studies highlight the value of both improving overall cardiovascular health and implementing targeted interventions addressing high-impact risk factors in AF.

Lifestyle factors often cluster together, resulting in a greater impact on cardiovascular health and disease progression [29]. Consistent with this, our moderation analysis showed that while improvements in cardiovascular health benefited all participants, those with multiple comorbidities or lower baseline LE8 scores experienced the greatest relative benefit. This is promising, given multimorbidity is highly prevalent in AF and known to complicate management [30]. In the RACE II study, patients with permanent AF and multiple comorbidities had significantly increased risks of all-cause mortality, heart failure, hospitalisations, and composite cardiovascular events compared with those with fewer conditions [31]. Evidence from population-based studies show that combinations of unhealthy behaviours often co-occur and markedly increase AF and cardiovascular risk. For example, individuals with all three risk behaviours—current smoking, heavy alcohol consumption, and physical inactivity—had a 22 % higher risk of AF [32]. Conversely, in a large nationwide cohort study of people with newly diagnosed AF (*n* = 208,662), a Healthy Lifestyle Score based on non-smoking, non-drinking, and regular exercise behaviours was associated with progressively lower MACE risk. Compared with those with none of these positive behaviours, having one associated with 21 % lower risk, two 35 % lower risk, and all three 42 % lower risk of outcomes including stroke, myocardial infarction, and hospitalisation for heart failure. These benefits were consistent across clinical subgroups and independent of anticoagulant use [33].

Lifestyle modification influences AF risk and burden through several key physiological mechanisms [34]. Obesity promotes atrial dilation, fibrosis, and autonomic dysfunction, while weight loss reduces inflammation, blood pressure, and improves metabolic health [35]. Physical activity enhances autonomic balance and reduces atrial inflammation, though excessive endurance training may promote atrial fibrosis and heightened vagal tone, highlighting the need for balanced exercise recommendations [36]. Diets high in saturated fats and refined sugars contribute to insulin resistance and oxidative stress [37], whereas cardioprotective diets—such as the Mediterranean and DASH diets—can improve metabolic profiles and oxidative stress [38]. Chronic alcohol consumption alters atrial electrophysiology, contributes to atrial remodelling, and increases left atrial pressure, while also promoting weight gain and hypertension [39]. Smoking is associated with persistent inflammation and oxidative stress; conversely, cessation is linked to lower AF risk [40,41]. Finally, epigenetic age has been shown to partly mediate lower cardiovascular disease risk with higher LE8 scores (i.e., decelerated biological ageing) [42]. These mechanisms emphasise the critical role of lifestyle in modulating the pathophysiological processes that underlie the AF substrate.

Guideline recommendations align closely with these insights. The ESC guidelines for AF management, through the AF-CARE pathway [43], and the 2023 ACC/AHA/ACCP/HRS guidelines [44], both place strong emphasis on aggressive comorbidity and risk factor management as a central pillar alongside traditional rhythm and rate control strategies [45]. Notably, the ESC AF-CARE pathway explicitly begins with comorbidity and risk factor optimisation, highlighting its foundational role in AF management. Similarly, the 2023 US guidelines highlight lifestyle and risk factor modification as a key strategy to prevent AF onset, slow its progression, and reduce complications [44]. Both sets of evolving guidelines increasingly recognise that management must extend beyond arrhythmia control to integrated, upstream interventions addressing modifiable risk factors. A recent narrative review also advocated for the integration of ExCR and structured lifestyle management within comprehensive AF care [46].

Our findings strongly support this prioritisation. We observed that better cardiovascular health—as reflected by higher LE8 scores—was significantly associated with lower risks of all-cause mortality and MACE. Interestingly, those with multiple comorbidities experienced the greatest relative benefit. These results directly reinforce the guideline emphasis on placing comorbidity and lifestyle optimisation at the forefront of AF care, demonstrating for the first time, that such interventions may be particularly impactful in high-risk AF populations.

## 5 Strengths and limitations

This study leveraged the UK Biobank, a large, well-characterised, population-based cohort, enabling robust investigation of the American Heart Association’s LE8 cardiovascular health score in individuals with AF. The large sample size allowed detailed stratified analyses by age and multimorbidity, non-linear modelling, landmark analyses, and competing risk approaches. To our knowledge, this is the first study to evaluate both overall LE8 and its individual components in relation to all-cause mortality and MACE in an AF population, providing novel insights with direct clinical relevance.

Several limitations should be acknowledged. First, as an observational analysis, causal inference cannot be established, and the population attributable fraction (PAF) and potential impact fraction (PIF) estimates should be interpreted as hypothetical, scenario-based measures under the assumptions of the modelling framework. These analyses rely on observational, baseline-only data and therefore assume no residual confounding, accurate exposure measurement, and stability of cardiovascular health behaviours during follow-up. Although extensive covariate adjustment was undertaken, residual confounding cannot be excluded, particularly from unmeasured factors such as frailty, functional status, or subclinical disease, which may influence both cardiovascular health behaviours and clinical outcomes.

Second, cardiovascular health metrics were assessed at a single baseline time point and were not updated during follow-up. Several LE8 components, including physical activity, diet, sleep, and smoking status, were self-reported, which may introduce measurement error and the potential for reverse causation, whereby poorer underlying health influences reported behaviours. In addition, several biological components of the LE8 score may be influenced by pharmacological treatment, which may reflect access to care or disease severity rather than lifestyle alone. Together, these factors may contribute to exposure misclassification and attenuation of observed associations.

Third, the PAF estimates were derived from non-linear spline-based hazard models and are inherently model-dependent. Similarly, PIF simulations assumed that a 20-point improvement in the LE8 score exerts a uniform effect across the score distribution, which may oversimplify the heterogeneous modifiability and non-linear relationships between individual LE8 components and risk. In addition, PIF analyses attributing impact to individual LE8 components should not be interpreted as independent or additive effects, as lifestyle and biological factors within the LE8 construct are likely correlated and may act through shared or overlapping pathways. These analyses are therefore best viewed as illustrative of potential population-level impact rather than causal effects of individual components.

Fourth, moderation analyses should be interpreted with caution. Although statistically significant interactions were observed for age and multimorbidity, these reflect heterogeneity in the strength of associations on the multiplicative (hazard ratio) scale rather than large differences in absolute risk. Given the large sample size, relatively modest differences in association strength may reach statistical significance, and subgroup findings should therefore not be overstated.

In addition, the dietary component of the LE8 score was adapted to reflect the dietary data available in the UK Biobank and therefore does not fully capture the American Heart Association Healthy Eating Index. While this approach aligns with UK and European dietary guidelines, it may affect the construct validity of the composite LE8 score, in addition to limiting comparability with studies using the full AHA-defined dietary metric.

The LE8 score also assigns equal weight to all eight components, despite evidence that individual lifestyle and biological factors may contribute differently to cardiovascular risk. This equal weighting may oversimplify the relative importance of specific components and should be considered when interpreting both the composite LE8 score and component-level analyses.

Finally, the UK Biobank cohort is not fully representative of the general population, with under-representation of certain ethnic and socioeconomically deprived groups, which may limit the generalisability of the findings.

## 6 Conclusion

In summary, a healthier lifestyle, as reflected by a higher LE8 score, was associated with a lower risk of all-cause mortality and MACE in people with AF over a 10-year follow up. Moreover, PIF analyses highlighted that increasing the LE8 through improvement in one component, could improve clinical outcomes. Component-level analyses identified diet, smoking, blood pressure, and BMI as the most influential factors, suggesting these may be particularly effective targets for intervention. Overall, our findings emphasise the importance of targeting modifiable lifestyle behaviours to reduce cardiovascular risk and mortality in individuals with AF.

**Central illustration**

**Figure:**
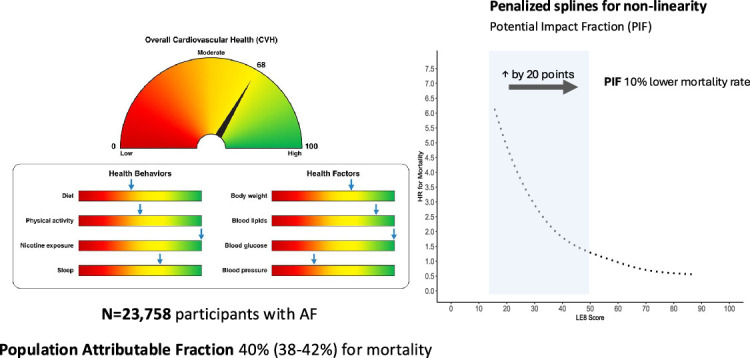
Among 23,758 participants with AF followed for 10 years, higher CVH scores were associated with progressively lower risks of all-cause mortality and major adverse cardiovascular events (MACE) in a non-linear, graded manner. Compared with the lowest CVH quartile, the highest quartile was associated with a 39% lower risk of all-cause mortality (HR 0.61, 95% CI 0.56–0.67) and a 38% lower risk of MACE (HR 0.62, 95% CI 0.58–0.67; both p < 0.001). Associations varied by age and multimorbidity. Simulation analyses suggested that improving CVH could reduce all-cause mortality by 10% and MACE by 7%, with diet, smoking, blood pressure, and body mass index contributing most to the potential risk reduction.

## Contributors

BB and CF conceived the study. BB performed the data analysis. CF drafted the manuscript. BB, CF, HJ and LF contributed to interpretation of results, critical manuscript revisions, and approved the final version for submission.

## Funding

No specific funding was received for this work.

**Guarantor:** BB accepts full responsibility for the work, had access to the data, and controlled the decision to publish.

## Patient and public involvement

Two PPIE workshops were undertaken by BB with 24 patients with AF who helped inform the study and dissemination materials. The study used existing data from the UK Biobank, a resource that involves ongoing public engagement and participant involvement at a resource-wide level.

## Ethical approval

Ethical approval was granted by the Northwest Multi-Centre Research Ethics (REC reference: 21/NW/0157). All participants provided written informed consent at baseline for data collection, follow-up, and linkage to health records.

**Data availability statement** The data underlying this article are available from the UK Biobank (www.ukbiobank.ac.uk) but restrictions apply.

## Declaration of competing interest

The authors declare the following financial interests/personal relationships which may be considered as potential competing interests:

BB has received investigator-initiated research funding from BMS/Pfizer and Huawei EU unrelated to this work.

## Appendix Supplementary materials

Image, application 1
