# Supplementary material for: Lifes essential 8 score and 10-year cardiovascular outcomes in atrial fibrillation: A UK biobank analysis with simulated lifestyle improvement
Source: Am J Prev Cardiol. 2025 Dec 29;25:101399. doi: 10.1016/j.ajpc.2025.101399 (PMC12856153; doi:10.1016/j.ajpc.2025.101399)
Supplement: Supplementary file 1 [file mmc1.docx]

**Supplementary Material**

**Supplement 1. Operationalisation of Life’s Essential 8 (LE8) Components**

A modified LE8 score (0-100) was derived from UK Biobank baseline data. Each component was scored according to American Heart Association (AHA) recommendations where possible, with adaptations made based on available variables.

| **Health Metric** | **UK Biobank Source (Field ID)** | **Measurement/Definition** | **Scoring** | |
| --- | --- | --- | --- | --- |
|  |  |  | **Points** | **Status** |
| **Smoking** | Self-report: current/past smoking (1239, 1249, 20116) | Never, former, current | 100  50  0 | Never = ideal  Former = intermediate  Current = poor |
| **Body mass index** | Measured height & weight (21001) | Body weight (kg) divided by height squared (m^2^) | 100  70  30  15  0 | <25 kg/m^2^  25.0 to 29.9 kg/m^2^  30.0 to 34.9 kg/m^2^  35.0 to 39.9 kg/m^2^  ≥40.0 kg/m^2^ |
| **Physical activity** | IPAQ-derived questionnaire (884, 904, 914) | Minutes/week moderate-to-vigorous physical activity | 100  90  80  60  50  40  20  0 | ≥1000 MET/min/week  ≥600 to <1000 MET/min/week  ≥480 to <600 MET/min/week  ≥360 to <480 MET/min/week  ≥240 to <360 MET/min/week  ≥120 to <240 MET/min/week  ≥4 to <120 MET/min/week  0 MET/min/week |
| **Cholesterol (non-HDL)** | Blood biochemistry (30780, 30760, 30690) | Calculated: Plasma total and HDL cholesterol | 100  60  40  20  0 | <130 mg/dl  ≥130 to 160 mg/dl  ≥160-190 mg/dl  ≥190 to 220 mg/dl  ≥220 mg/dl |
| **Blood pressure** | Measured systolic/diastolic BP (4080, 4079) | Mean of 2 seated measures | 100  75  50  25  0 | <120 & <80 mmHg  ≥120 to 129 & <80 mmHg  ≥130 to 139 or ≥80 to 89 mmHg  ≥140 to 159 or ≥90 to 99 mmHg  ≥160 or ≥100 mmHg |
| **HbA1c** | Blood biochemistry (30750) | Appropriately measured systolic and diastolic blood pressure (mmol/mol) | 100  60  40  30  20  10  0 | <5.7 %  ≥5.7 to 6.4 %  6.4 to <7.0%  ≥7.0 to 7.9%  ≥8.0 to 8.9%  ≥9.0 to 9.9%  ≥10.0% |
| **Sleep** | Self-report sleep duration (1160) | Hours per night | 100  90  70  40  20  0 | ≥7 to <9 h/day  ≥9 to <10 h/day  ≥6 to <7 h/day  ≥5 to <6 or ≥10 h/day  ≥4 to <5 h/day  <4 h/day |

**Supplement 2. Adapted Healthy Diet Score**

| **Diet Component** | **Criteria (UK/European guidelines)** | **Scoring** |
| --- | --- | --- |
| Processed meat | ≤1 serving/week | 0 = unhealthy; 1 = healthy |
| Red meat | ≤2 servings/week | 0 = unhealthy; 1 = healthy |
| Fish (total) | ≥2 servings/week (incl. oily fish) | 0 = unhealthy; 1 = healthy |
| Alcohol | ≤14 units/week (men), ≤7 units/week (women) | 0 = unhealthy; 1 = healthy |
| Spread type  Cereal intake | Non-hydrogenated / unsaturated (e.g. olive/veg oil)  Wholegrain preference | 0 = unhealthy; 1 = healthy  0 = unhealthy; 1 = healthy |
| Salt added to food  Water intake  Fruit & vegetables | Never/rarely  ≥5 glasses/day  ≥5 portions/day | 0 = unhealthy; 1 = healthy  0 = unhealthy; 1 = healthy  0 = unhealthy; 1 = healthy |

**Healthy diet scoring**

Briefly, a total of 9 food items were used to create the diet score, including processed meat, red meat, total fish, alcohol, spread type, cereal intake, salt added to food, water, and fruits and vegetables. Each food item was dichotomized as meeting or not meeting recommendations as suggested by the UK and European dietary guidelines. Participants were given 1 point for each unhealthy category. Finally, a diet score ranging from 0 (healthiest) to 9 (least healthy) was derived by summing the points for each participant. This was then split into quartiles for LE8 scoring.

**Supplement 3. ICD-10 Codes Used to Ascertain Outcomes**

| **Outcome** | **ICD-10 Codes** | **Source** |
| --- | --- | --- |
| Ischaemic Heart Disease (IHD) | I20–I25 | Linked HES/SMR01 records |
| Myocardial Infarction (MI) | I21–I23 | Linked HES/SMR01 records |
| Stroke | I60, I61, I63, I64 | Linked HES/SMR01 records |
| Heart Failure (HF) | I50.0, I50.1, I50.9 | Linked HES/SMR01 records |
| All-cause mortality | Death certificate data | NHS Information Centre / NHS Central Register |
